# *Prochlorococcus* Exudate Stimulates Heterotrophic Bacterial Competition with Rival Phytoplankton for Available Nitrogen

**DOI:** 10.1128/mbio.02571-21

**Published:** 2022-01-11

**Authors:** Benjamin C. Calfee, Liz D. Glasgo, Erik R. Zinser

**Affiliations:** a Department of Microbiology, University of Tennessee, Knoxville, Tennessee, USA; University of California, Irvine

**Keywords:** *Prochlorococcus*, *Synechococcus*, *Alteromonas*, competition, nitrogen limitation, resource competition

## Abstract

The marine cyanobacterium *Prochlorococcus* numerically dominates the phytoplankton community of the nutrient-limited open ocean, establishing itself as the most abundant photosynthetic organism on Earth. This ecological success has been attributed to lower cell quotas for limiting nutrients, superior resource acquisition, and other advantages associated with cell size reduction and genome streamlining. In this study, we tested the prediction that *Prochlorococcus* outcompetes its rivals for scarce nutrients and that this advantage leads to its numerical success in nutrient-limited waters. Strains of *Prochlorococcus* and its sister genus *Synechococcus* grew well in both mono- and cocultures when nutrients were replete. However, in nitrogen-limited medium, *Prochlorococcus* outgrew *Synechococcus* but only when heterotrophic bacteria were also present. In the nitrogen-limited medium, the heterotroph Alteromonas macleodii outcompeted *Synechococcus* for nitrogen but only if stimulated by the exudate released by *Prochlorococcus* or if a proxy organic carbon source was provided. Genetic analysis of *Alteromonas* suggested that it outcompetes *Synechococcus* for nitrate and/or nitrite, during which cocultured *Prochlorococcus* grows on ammonia or other available nitrogen species. We propose that *Prochlorococcus* can stimulate antagonism between heterotrophic bacteria and potential phytoplankton competitors through a metabolic cross-feeding interaction, and this stimulation could contribute to the numerical success of *Prochlorococcus* in nutrient-limited regions of the ocean.

## INTRODUCTION

The phytoplankton community occupying the vast majority of the sunlit ocean experiences chronic nutrient limitation (1–4). Depending on the location, the limiting nutrients include nitrogen, phosphorus, iron, and other metals. While the diversity of phytoplankton in these regions can be quite high, numerical superiority is often achieved by a single genus of cyanobacteria, *Prochlorococcus* (105). The most abundant photosynthetic organism in the ocean, *Prochlorococcus* can grow to populations that exceed 100,000 cells mL^−1^, besting its competitors by orders of magnitude in many instances (5–8).

The reasons underpinning the numerical dominance of *Prochlorococcus* in nutrient-limited waters have not been fully elucidated, but several distinguishing features of this unusual cyanobacterium have been implicated. *Prochlorococcus* has the smallest cell and genome size for a photoautotroph, which collectively lower the cell quota for nitrogen, iron, and phosphorus (9–12). The phosphorus quota is further reduced by the replacement of phospholipids with sulfolipids as the predominant membrane lipids (13, 14). Additional means of economy (10, 15–17) may further contribute to the ability of *Prochlorococcus* to reproduce at a lower cost than its competitors under nutrient-limited conditions.

A reduction in cell size is thought to provide *Prochlorococcus* with the additional advantage of superior nutrient acquisition (18). Lomas et al. noted that when normalized to the cell quota, *Prochlorococcus* had a higher affinity for phosphate than *Synechococcus* and picoeukaryotic phytoplankton (19). Notably, resource competition theory applied to global ocean simulations predicted the numerical domination of the oligotrophic regions by analogs of *Prochlorococcus*, which could draw nutrients down to concentrations that cannot be accessed by their competitors (20–22).

Despite the net loss of genes through streamlining, the diversity within the genus *Prochlorococcus* is high and believed to contribute to the numerical dominance of *Prochlorococcus* by facilitating niche expansion. Phylogenetically distinct clades, termed ecotypes, exist within the genus and have demonstrated different optima for temperature, light intensities, and nutrient utilization that correlate with their environmental distributions (23–31). Notably, within these ecotypes, subecotypes have been found with their own distinct ecologies, suggesting that the open-ocean niche is finely partitioned through environmental influences on *Prochlorococcus* evolution (32–34).

A final contributor to the ecological success of *Prochlorococcus* may be the help that it receives from the microbial community. All known genomes of *Prochlorococcus* lack the gene encoding the hydrogen peroxide scavenger catalase (35–37). The loss of catalase is believed to improve the growth efficiency by reducing cell quotas for iron and/or nitrogen, but it leaves cells highly susceptible to oxidative damage from environmental sources of hydrogen peroxide (12, 36, 38). *Prochlorococcus* survives this threat because it is cross-protected by cooccurring catalase-positive “helpers” such as Alteromonas macleodii, a heterotroph frequently coisolated with *Prochlorococcus* (12, 35, 39). Alteromonas macleodii rapidly scavenges extracellular H_2_O_2_, causing changes in gene expression and promoting the growth of cocultured *Prochlorococcus* under conditions that would otherwise be lethal (35, 40–42).

The physiological and genetic features of *Prochlorococcus* all predict a competitive advantage over rival phytoplankton under nutrient-limited conditions, and this advantage may contribute significantly to its ecological success in the oligotrophic ocean. In this work, we sought direct evidence that *Prochlorococcus* could achieve numerical superiority over a key rival, *Synechococcus*. We focused our study on nitrogen-limiting conditions simulating the North Pacific Subtropical Gyre (NPSG) (43), where *Prochlorococcus* outnumbers *Synechococcus* and other rival phytoplankton by an order of magnitude or more (6, 8, 44). We found that competition for nitrogen explained the differences in *Prochlorococcus* and *Synechococcus* abundances but only through the presence and specific activity of marine heterotrophic bacteria fed by *Prochlorococcus*-derived carbon. As these outcomes matched previous predictions of *Prochlorococcus* success, we argue that conditions such as the ones examined could provide important insight into the global ecology of *Prochlorococcus*.

## RESULTS

### *Prochlorococcus* outcompetes *Synechococcus* in the presence of heterotrophs.

**FIG 1:**
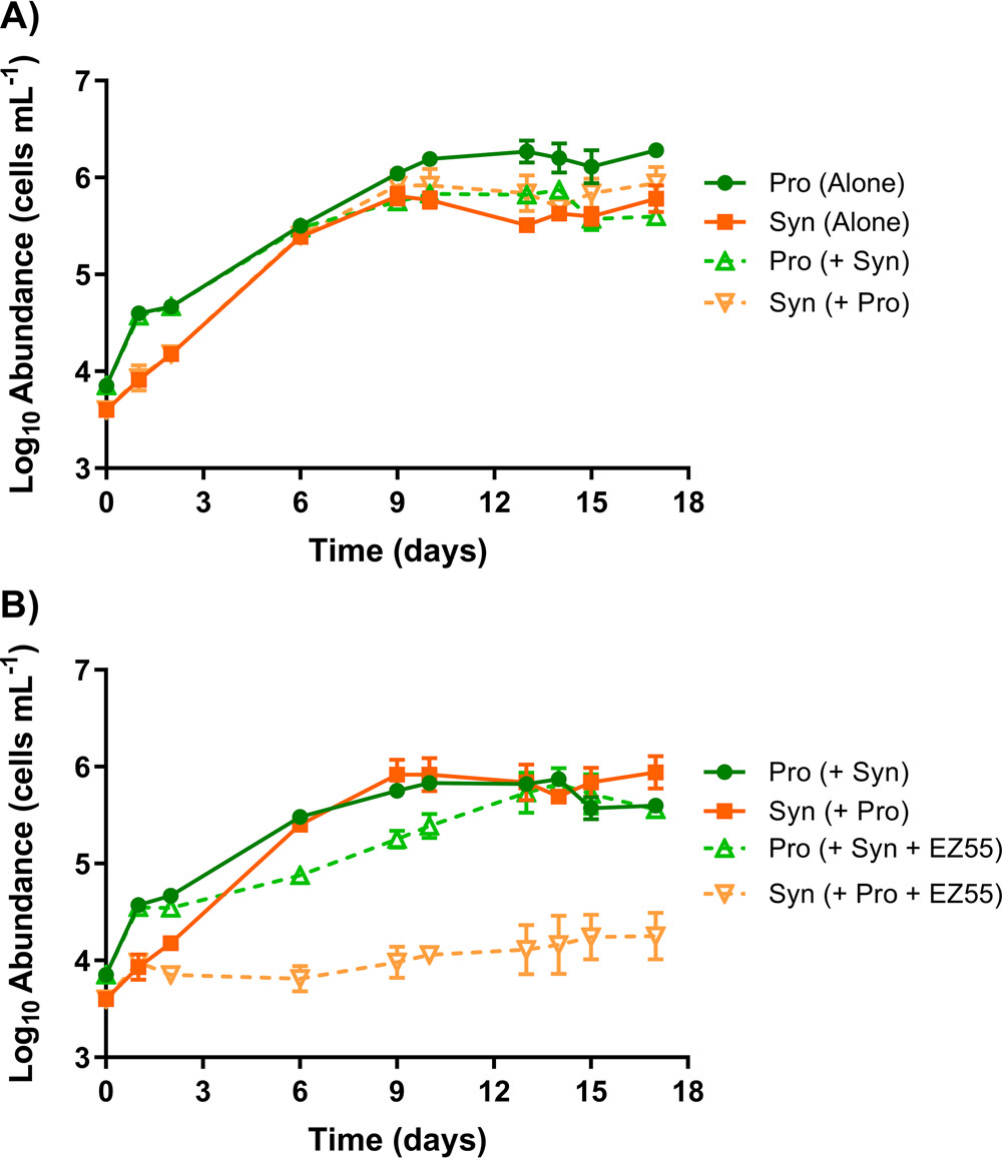
Mono-, co-, and tripartite culture competition. The growth of *Prochlorococcus* strain MIT9215 (Pro) and *Synechococcus* strain WH7803 (Syn) in AMP-MN artificial seawater medium in monoculture (A), cyanobacterial coculture (the same data are shown in panels A and B), and a tripartite culture with Alteromonas macleodii strain EZ55 (B) was determined. Error bars represent 1 standard deviation of the geometric mean (*n* = 3).

Cyanobacterial growth in mono- and cocultures was assessed in low-nitrogen medium (artificial medium for *Prochlorococcus* minus nitrogen [AMP-MN]), an artificial seawater medium lacking N amendment and containing approximately 0.164 μM residual bioavailable N (see Materials and Methods; see also Fig. S1 in the supplemental material). *Prochlorococcus* sp. strain MIT9215 reached a higher maximum abundance in monoculture than in coculture with *Synechococcus* sp. strain WH7803, suggesting that competition in coculture caused a slight but significant reduction in the MIT9215 cell yield (Fig. 1A) (*P* < 0.0001). WH7803 maximum abundances did not differ between monoculture and coculture with MIT9215 (Fig. 1A) (*P* = 0.2754).

10.1128/mBio.02571-21.1 FIG S1 Determination of bioavailable nitrogen in AMP-MN. The growth of *Prochlorococcus* strain MIT9215 (A) and *Synechococcus* strain WH7803 (B) in AMP-MN artificial seawater medium amended with various concentrations of ammonium was determined. Error bars represent 1 standard deviation of the geometric mean (*n* = 3). Download FIG S1, TIF file, 1.5 MB. Copyright © 2022 Calfee et al. 2022 Calfee et al. https://creativecommons.org/licenses/by/4.0/ This content is distributed under the terms of the Creative Commons Attribution 4.0 International license.

The addition of the marine heterotrophic bacterium Alteromonas macleodii strain EZ55 dramatically changed the outcome for the *Synechococcus*-*Prochlorococcus* cocultures (Fig. 1B). While the *Prochlorococcus* strain MIT9215 growth rate declined moderately, the addition of EZ55 to the coculture resulted in a nearly total loss of growth for *Synechococcus* strain WH7803 (*P* = 0.0018). In this AMP-MN medium, the EZ55 heterotroph grew rapidly to ∼10^6^ cells mL^−1^, regardless of whether cyanobacteria were present (see below), indicating growth on trace contaminating organic carbon in the medium. The presence of the heterotroph in this nitrogen-limited medium thus shifted the phytoplankton community structure to one resembling open-ocean communities, with *Prochlorococcus* being numerically dominant over its rival *Synechococcus*.

**FIG 2:**
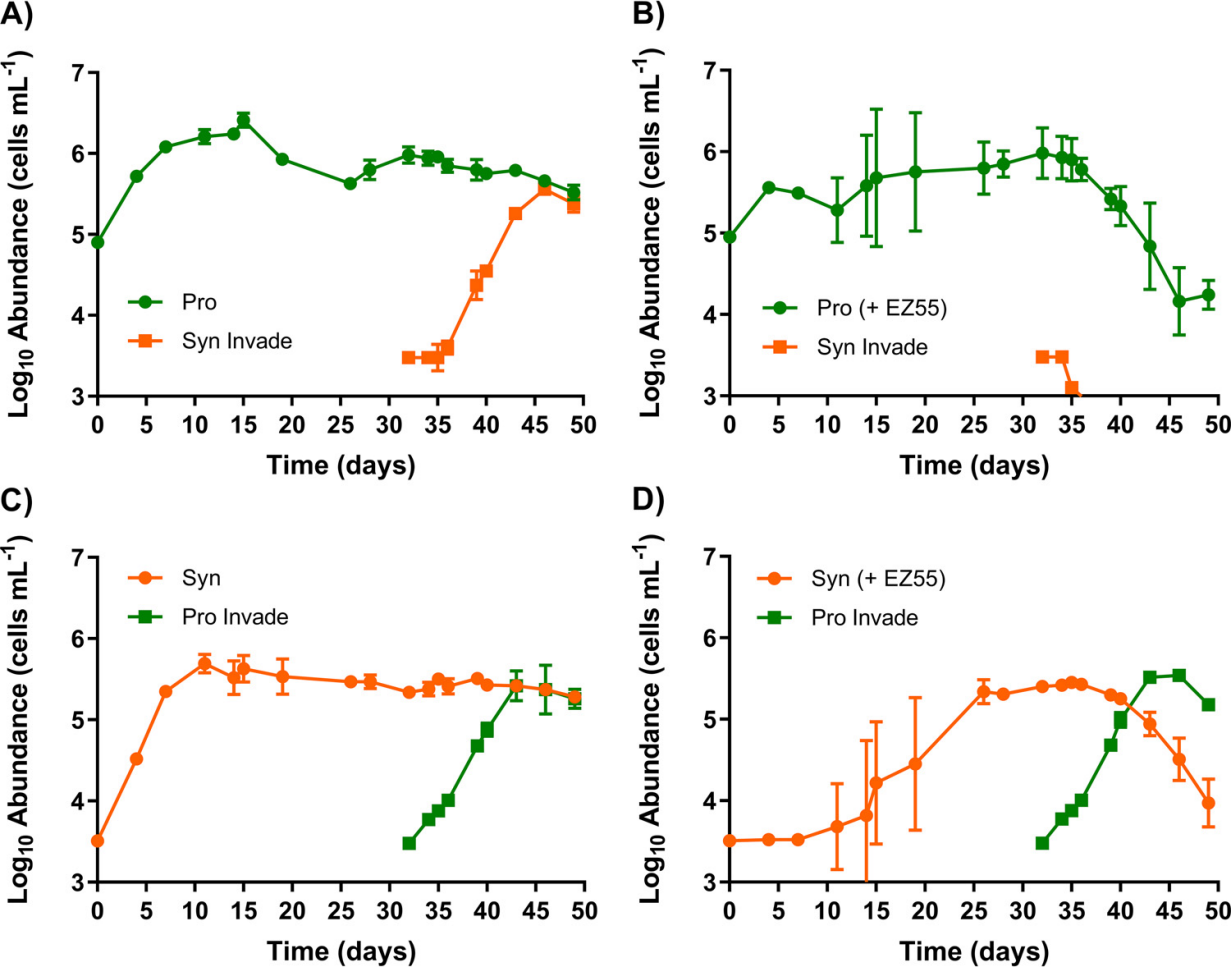
Invasion assay. The growth of *Prochlorococcus* strain MIT9215 (A and B) and *Synechococcus* strain WH7803 (C and D) in AMP-MN artificial seawater medium with and without Alteromonas macleodii strain EZ55 was determined. On day 32, cultures of the cyanobacteria without *Alteromonas* were inoculated as a minority into the cultures of the rival cyanobacterium with and without *Alteromonas* to assess the ability to invade. Error bars represent 1 standard deviation of the geometric mean (*n* = 3).

The dynamics of resource competition were further investigated by challenging the cyanobacterial strains to invade established populations of their competitors when rare. At day 32 of growth in AMP-MN, a small inoculum (∼3,000 cells mL^−1^) from *Synechococcus* strain WH7803 monocultures was added to cultures of *Prochlorococcus* strain MIT9215 with or without Alteromonas macleodii strain EZ55; reciprocally, MIT9215 monocultures were inoculated into cultures of WH7803 with or without EZ55. WH7803 cells were able to invade MIT9215 monocultures after a few days’ lag and reach an almost equal abundance over the next 17 days (Fig. 2A). However, WH7803 failed to grow in MIT9215 cultures when EZ55 was present, dropping below the limit of detection shortly after inoculation (Fig. 2B).

In the reciprocal invasion assay, *Prochlorococcus* strain MIT9215 rapidly grew when inoculated into the *Synechococcus* strain WH7803 monoculture, with both organisms coexisting at equal abundances (Fig. 2C). In the presence of Alteromonas macleodii strain EZ55, MIT9215 was still able to invade a culture of WH7803 (Fig. 2D). Interestingly, with EZ55 present, the MIT9215 population displaced WH7803 as the majority phytoplankter in the culture: WH7803 exhibited a dramatic decline in abundance (Fig. 2D) that was not observed when EZ55 was absent (Fig. 2C). Thus, independent of the starting ratios or cell concentrations, the presence of the EZ55 heterotroph favored the growth of *Prochlorococcus* over *Synechococcus* when cultured in nitrogen-limited media.

### *Prochlorococcus* exudate drives heterotroph N competition with *Synechococcus*.

**FIG 3:**
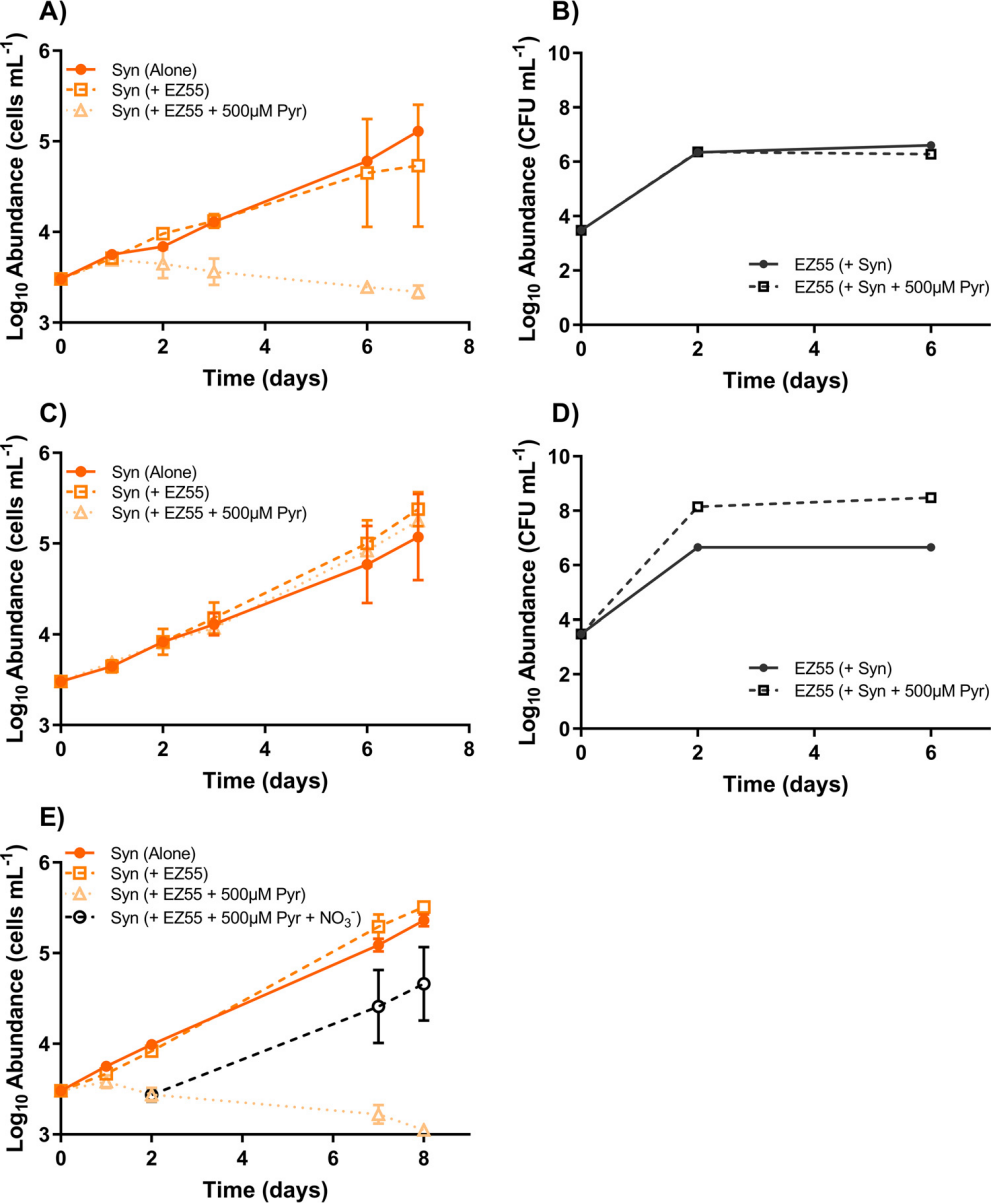
*Synechococcus*-*Alteromonas* interactions. The growth of *Synechococcus* strain WH7803 (A, C, and E) and Alteromonas macleodii strain EZ55 (B and D) in AMP-MN (A, B, and E) and AMP-A (C and D) artificial seawater media in monoculture, coculture, and coculture with the addition of 500 μM sodium pyruvate (Pyr) was determined. Cocultures were also amended with 500 μM sodium pyruvate and 800 μM sodium nitrate to demonstrate growth rescue by nutrient addition (E). Error bars represent 1 standard deviation of the geometric mean (*n* = 3).

**FIG 4:**
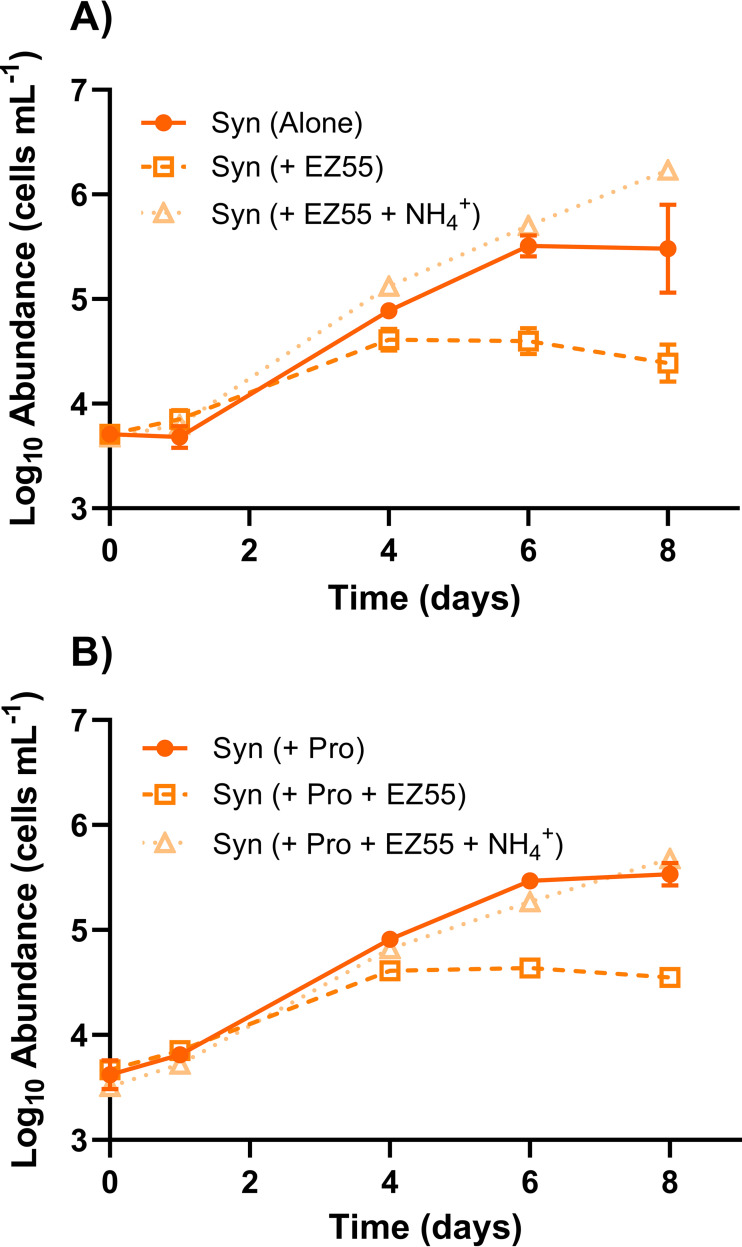
*Synechococcus*-*Alteromonas* coculture in *Prochlorococcus*-conditioned AMP-MN. The growth of *Synechococcus* strain WH7803 in monoculture or coculture with Alteromonas macleodii strain EZ55 with or without 400 μM NH_4_^+^ in AMP-MN artificial seawater medium preconditioned by the growth of *Prochlorococcus* strain MIT9215, after the removal of these *Prochlorococcus* cells via filtration (A) or when they were allowed to remain in the media (B), was determined. Error bars represent 1 standard deviation of the geometric mean (*n* = 3).

Critically, the inhibitory effect of Alteromonas macleodii strain EZ55 on *Synechococcus* strain WH7803 growth was absent if the *Prochlorococcus* MIT9215 strain was not included. WH7803 showed no significant difference in growth between mono- and cocultures with EZ55 in AMP-MN during exponential growth (Fig. 3A) (*P* = 0.91). This outcome suggested that *Prochlorococcus* may be secreting a factor(s) that stimulates the competition of EZ55 for a resource(s) shared by WH7803. To test this, EZ55 and WH7803 were placed in coculture competition in medium preconditioned by MIT9215. Whether MIT9215 cells were removed (via filtration) prior to competition (Fig. 4A) or remained in the medium (Fig. 4B and Fig. S2), the outcome was the same, and the WH7803 maximal abundance was reduced by an order of magnitude when cocultured with EZ55 compared to its inoculation alone in MIT9215-conditioned medium. As shown in Fig. 3A, this growth differential was not observed in the same growth medium when MIT9215 was absent and did not precondition the medium.

10.1128/mBio.02571-21.2 FIG S2 *Prochlorococcus* growth during the medium preconditioning experiment. The growth of *Prochlorococcus* strain MIT9215 in preconditioned AMP-MN artificial seawater medium after inoculation of *Synechococcus* strain WH7803 and Alteromonas macleodii strain EZ55 was determined. Error bars represent 1 standard deviation of the geometric mean (*n* = 3). Download FIG S2, TIF file, 1.2 MB. Copyright © 2022 Calfee et al. 2022 Calfee et al. https://creativecommons.org/licenses/by/4.0/ This content is distributed under the terms of the Creative Commons Attribution 4.0 International license.

We next considered two hypotheses for the *Prochlorococcus*-driven loss of *Synechococcus* strain WH7803 growth in the presence of Alteromonas macleodii strain EZ55: *Prochlorococcus* is driving EZ55 to either compete for limited resources or produce a factor that is toxic to WH7803. Carbon and nitrogen amendment studies favored the former over the latter hypothesis.

*Prochlorococcus* releases a large fraction of fixed carbon as dissolved organic carbon during nitrogen-limited growth (45), so we reasoned that this excess source of carbon and energy could be energizing Alteromonas macleodii strain EZ55 to compete with *Synechococcus* for nitrogen in this nitrogen-limited medium. Pyruvate was examined as a proxy for the *Prochlorococcus* exudate and, like the exudate, allowed EZ55 to prevent the growth of *Synechococcus* strain WH7803 (Fig. 3A). Notably, in tripartite cultures, the addition of pyruvate (Fig. S3) further contributed to WH7803 reduction without an apparent effect on *Prochlorococcus* strain MIT9215.

10.1128/mBio.02571-21.3 FIG S3 Effect of pyruvate addition on tripartite outcomes. The growth of *Prochlorococcus* strain MIT9215, *Synechococcus* strain WH7803, and Alteromonas macleodii strain EZ55 in a tripartite culture in AMP-MN artificial seawater medium with and without the addition of 500 μM sodium pyruvate was determined. Error bars represent 1 standard deviation of the geometric mean (*n* = 3). Download FIG S3, TIF file, 1.3 MB. Copyright © 2022 Calfee et al. 2022 Calfee et al. https://creativecommons.org/licenses/by/4.0/ This content is distributed under the terms of the Creative Commons Attribution 4.0 International license.

In AMP-MN medium, which is identical to artificial medium for *Prochlorococcus* autoclaved (AMP-A) except for the omission of nitrogen addition (see Materials and Methods), nitrogen is the limiting resource for both *Prochlorococcus* and *Synechococcus* (Fig. S1A and B); other nutrients were provided in excess. As such, we reasoned that if Alteromonas macleodii strain EZ55 was restricting the growth of *Synechococcus* strain WH7803, it was likely via competition for nitrogen. Consistently, the addition of excess nitrogen to the medium as either NH_4_^+^ or NO_3_^−^ restored the ability of WH7803 to grow in the presence of pyruvate or exudate-stimulated EZ55, whether at the onset of cocultivation (Fig. 3C and Fig. 4A and B) or after WH7803 had ceased growth for several days (Fig. 3E). Notably, in these coculture studies, pyruvate additions enabled EZ55 to grow to levels several orders of magnitude higher when nitrogen was in excess (Fig. 3D) but not when nitrogen was limiting (Fig. 3B), suggesting that inhibition by EZ55 requires excess carbon relative to nitrogen.

### Nitrogen competition in three-member cocultures.

**FIG 5:**
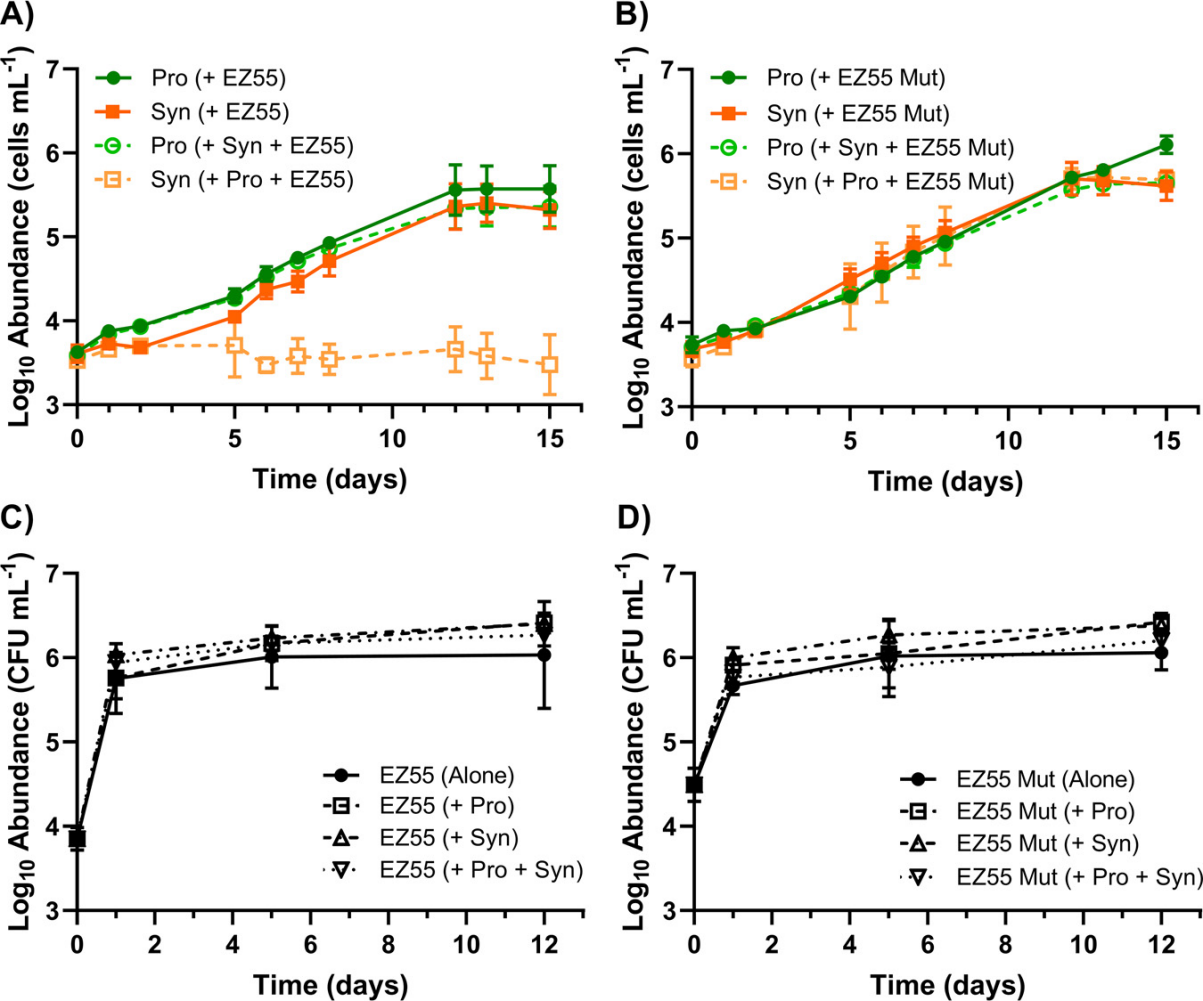
Effect of *Alteromonas* nitrate utilization mutant on tripartite outcomes. (A and B) Growth of *Prochlorococcus* strain MIT9215 and *Synechococcus* strain WH7803 in AMP-MN artificial seawater medium in a coculture and tripartite culture with WT Alteromonas macleodii strain EZ55 (A) or the Alteromonas macleodii strain EZ55 *nirB* mutant (Mut) (B). (C and D) Abundance of heterotrophs in each treatment for the WT (C) and the mutant (D). Error bars represent 1 standard deviation of the geometric mean (*n* = 3).

While the concentration of total bioavailable N in AMP-MN has been established (Fig. S1), the constituent N species are not known. We hypothesized that while the *Prochlorococcus* strain consumes NH_4_^+^, the *Synechococcus* and heterotroph strains compete for a residual N resource that *Prochlorococcus* cannot utilize but that the other two can, namely, NO_3_^−^ or NO_2_^−^ (46). To test this hypothesis, we generated a transposon insertion mutant of Alteromonas macleodii strain EZ55 with a loss-of-function mutation in the *nirB* gene (nitrite reductase large subunit). The *nirB* mutant cannot utilize nitrate or nitrite as a nitrogen source and, unlike the wild type (WT) (Fig. 5A), cannot prevent the growth of *Synechococcus* strain WH7803 in tripartite cultures with *Prochlorococcus* strain MIT9215 (Fig. 5B). The *nirB* mutation did not impact the growth of the *Alteromonas* strain (Fig. 5C and D), suggesting that this mutation prevented nitrogen competition without impacting overall growth. The inability of the EZ55 *nirB* mutant to restrict the growth of WH7803 suggests that NO_3_^−^/NO_2_^−^ was present in AMP-MN and that wild-type EZ55 is able to outcompete WH7803 for this resource (when activated by the *Prochlorococcus* exudate).

### Competition outcomes are robust with regard to genotype.

**FIG 6:**
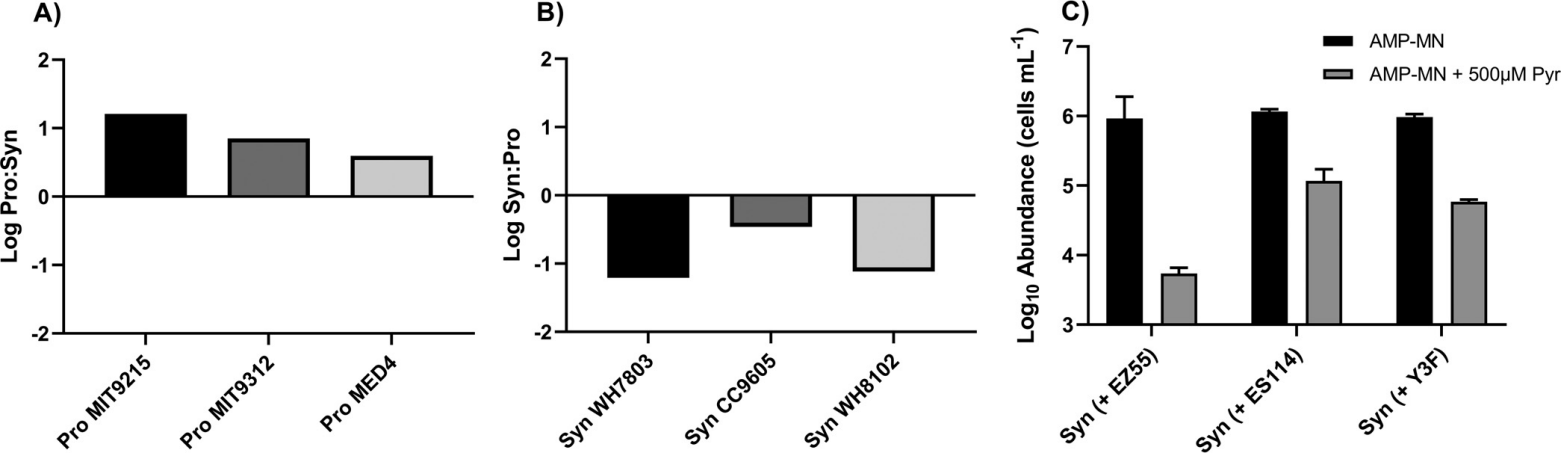
Effect of strain variability on competition outcome. (A and B) Comparison of log_10_ ratios of different *Prochlorococcus* (A) and *Synechococcus* (B) strains’ maximal abundances in tripartite cultures with Alteromonas macleodii strain EZ55 in AMP-MN artificial seawater medium. *Prochlorococcus* strains were cultured with *Synechococcus* strain WH7803 and EZ55 (A), and *Synechococcus* strains were cultured with *Prochlorococcus* strain MIT9215 and EZ55 (B). (C) Maximum abundances of *Synechococcus* strain WH7803 were also observed when cultured in AMP-MN or AMP-MN plus 500 μM sodium pyruvate with different marine heterotrophic bacteria. Error bars represent 1 standard deviation of the geometric mean (*n* = 3).

To determine the extent to which strain genotype impacts the outcomes of cocultivation, we modified the mixed-culture experiments by replacing *Prochlorococcus* strain MIT9215, *Synechococcus* strain WH7803, or Alteromonas macleodii strain EZ55 with different strains of *Prochlorococcus*, *Synechococcus*, or heterotrophic bacteria, respectively. Like MIT9215, high-light-adapted *Prochlorococcus* sp. strain MIT9312 or MED4 outcompeted WH7803 in the presence of EZ55 (Fig. 6A), and like WH7803, *Synechococcus* sp. strains CC9605 and WH8102 were outcompeted by MIT9215 in the presence of EZ55 (Fig. 6B).

As a final constraint on the *Synechococcus*-heterotroph coculture outcomes, different marine heterotrophic bacteria were substituted for Alteromonas macleodii strain EZ55: *Phaeobacter* sp. strain Y3F and Vibrio fischeri strain ES114. When grown in N-replete AMP-A with or without pyruvate or N-limited AMP-MN without pyruvate, coculturing with any of the three heterotrophs did not cause any significant deviation of the *Synechococcus* strain WH7803 maximal abundance compared to the monoculture control (Fig. S4A to C). However, as with EZ55, the addition of pyruvate to AMP-MN caused a reduction in the WH7803 maximal abundance when in coculture with YF3 or ES114 compared to either the monoculture control (Fig. S4D) (*P* < 0.0001) or cocultures in AMP-MN without pyruvate (Fig. 6C) (*P* < 0.0001). With the exception of ES114, all heterotrophs maintained steady long-term populations in AMP-MN regardless of amendments; ES114 declined steadily and maintained its starting abundance only with pyruvate addition (Fig. S4E to G).

10.1128/mBio.02571-21.4 FIG S4 *Synechococcus*-heterotroph coculture interactions. (A to D) Growth of *Synechococcus* strain WH7803 in AMP-A and AMP-MN artificial seawater media with and without 500 μM sodium pyruvate in monoculture and coculture with individual heterotrophs: Alteromonas macleodii strain EZ55, Vibrio fischeri strain ES114 (EZ87), and *Phaeobacter* sp. strain Y3F (EZ127). (E to G) Heterotroph abundances in all coculture treatments. Error bars represent 1 standard deviation of the geometric mean (*n* = 3). Download FIG S4, TIF file, 1.7 MB. Copyright © 2022 Calfee et al. 2022 Calfee et al. https://creativecommons.org/licenses/by/4.0/ This content is distributed under the terms of the Creative Commons Attribution 4.0 International license.

## DISCUSSION

In this study, we describe conditions under which the dominance of *Prochlorococcus* over rival phytoplankton is reproduced in culture. Importantly, we observed that *Prochlorococcus* outgrows *Synechococcus* under low-nitrogen conditions, simulating the North Pacific Subtropical Gyre, and only in the presence of heterotrophic bacteria, simulating the multitrophic mixed community of the ocean.

In the NPSG, where nitrogen is thought to limit growth (3, 4, 13, 47), *Prochlorococcus* can outnumber *Synechococcus* (and other members of the phytoplankton community) by several orders of magnitude (6, 8, 44). In these nitrogen-limited waters, heterotrophic bacteria can grow to between 300,000 and 500,000 cells mL^−1^ and outnumber phytoplankton (48–50). Our low-nitrogen culture medium recapitulated these trends: heterotrophs grew to an only slightly higher abundance of 10^6^ cells mL^−1^, and in tripartite cultures, the dynamics of the picocyanobacteria favored *Prochlorococcus* over *Synechococcus*, regardless of the relative starting abundances.

Our results suggest that *Prochlorococcus* acts indirectly, through a heterotroph intermediate, to dictate the growth outcome of its rival *Synechococcus* in low-nitrogen environments. In low-nitrogen, low-organic-carbon medium, *Prochlorococcus* scavenges a residual source(s) of nitrogen, apparently with a superior capability relative to *Alteromonas* and *Synechococcus*. *Alteromonas* can grow on residual organic carbon until it becomes growth arrested by a lack of carbon and energy. In this state, it is poised to compete for nitrogen but lacks the carbon and energy resources to do so unless fed by *Prochlorococcus*. Once fed, *Alteromonas* can begin to compete with *Synechococcus* for an alternative nitrogen source(s). The inability of a mutant *Alteromonas* strain lacking the capacity for NO_3_^−^/NO_2_^−^ utilization to arrest the growth of *Synechococcus* suggests that the competition involves one or both of these nitrogen species, resources that both *Synechococcus* and wild-type *Alteromonas* can utilize but that the strains of *Prochlorococcus* examined in this study cannot. Nitrate-utilizing strains of *Prochlorococcus* were recently isolated (51), and future studies in tripartite cultures with these strains could prove informative. In the paragraphs that follow, we unpack this model to discuss the key supporting evidence and identify unanswered questions.

Our study implicates the release of organic carbon by *Prochlorococcus* for the stimulation of *Alteromonas* to outcompete *Synechococcus* for nitrogen. Neither *Prochlorococcus* nor *Alteromonas* acting alone was sufficient to diminish the growth of *Synechococcus*, but when together in a tripartite community, they diminished *Synechococcus* growth.

Importantly, this effect was observed only when nitrogen was limiting in the medium; the addition of excess nitrogen was all that was needed to restore *Synechococcus* growth. The latter result also argues against the production of a growth-limiting substance by *Alteromonas* as the explanation for the growth arrest of *Synechococcus*.

The *Prochlorococcus* exudate was sufficient to stimulate N competition by *Alteromonas*, as was a proxy form of the *Prochlorococcus* exudate, pyruvate. *Prochlorococcus* exudes a large fraction of fixed carbon as dissolved organic matter (52–54), much of which is bioavailable to heterotrophic bacteria (55, 56). Recently, it was observed that *Prochlorococcus* can also release membrane vesicles (57), which may serve as complex nutrients for cooccurring heterotrophs. Critically, under nitrogen limitation, the release of dissolved organic matter by *Prochlorococcus* is exacerbated (45, 58). The specific form(s) of released organic carbon that stimulated *Alteromonas* competition for nitrogen in this study is not known, but it is rather curious that the *Synechococcus* exudate was not sufficient for this effect: bipartite cultures of *Alteromonas* and *Synechococcus* stably coexisted in low-N medium. *Synechococcus* is known to release organic carbon, and this release increases under nutrient limitation (59), so this distinction between *Prochlorococcus* and *Synechococcus* exudates warrants further investigation.

As with carbon, the nitrogen species involved in the tripartite interactions are not yet completely identified and could include both inorganic and organic sources for growth. Our artificial seawater medium lacked nitrogen amendment, but trace amounts of nitrogen from unknown sources could support microbial growth to 10^6^ cells mL^−1^. Due to the volatility of ammonia and reported cases of ammonia contamination in other systems (60), we suspect that it serves as a major component of the unamended medium. As the preferred nitrogen source for *Prochlorococcus* and most microbes, we suspect that ammonia is the primary nitrogen source consumed by *Prochlorococcus*, whether in mono- or mixed cultures. However, strain MIT9215 has the genetic potential to utilize urea as well (37, 46), so this species cannot be ruled out. Nitrate and/or nitrite is likely a component of the medium, as *Synechococcus* strain WH7803 can utilize nitrate or nitrite as a sole nitrogen source (46), and *Alteromonas* became unable to prevent *Synechococcus* growth when the nitrite/nitrate utilization pathway of the heterotroph was knocked out. While some strains of *Prochlorococcus* can utilize nitrite and nitrate (51), the ones assayed in this study could not. Whether or not the nitrate/nitrite-utilizing *Prochlorococcus* strains can also compete with *Synechococcus* for this resource could be resolved in future studies.

In the ocean, *Prochlorococcus* and *Synechococcus* compete for a variety of nitrogen sources, including organic forms such as amino acids (29, 61–65). In a 2019 study, Berthelot et al. observed that cooccurring populations of *Prochlorococcus*, *Synechococcus*, and photosynthetic picoeukaryotes in the N-limited North Pacific Subtropical Gyre all utilize ammonia, urea, and nitrate although to different extents (62).

While capable of sourcing their nitrogen from organic carbon molecules like amino acids, marine heterotrophs have been shown to also compete with phytoplankton for inorganic nitrogen in the form of ammonia or nitrate (66–69). Heterotrophs can account for 30% or more of inorganic nitrogen uptake at some locations (70, 71), and in some studies, inorganic nitrogen accounted for half or more of the total nitrogen acquired by heterotrophs (72, 73).

Importantly, the ability of heterotrophs to compete for inorganic nitrogen appears to be stimulated by organic carbon. Several studies by the Kirchman group and others noted the necessity for sufficient carbon for inorganic N uptake by bacteria (67, 68, 73–76). These results reflect the importance of C/N balance for heterotrophic growth, which has been recognized in studies of Escherichia coli and other heterotrophs. For Escherichia coli, carbon limitation depletes the tricarboxylic acid (TCA) cycle intermediate and key substrate for inorganic nitrogen assimilation, α-ketoglutarate (2-oxoglutarate) (77). Consequently, C-starved cells have diminished rates of ammonium assimilation and potentially other N utilization pathways (77). Notably, a recent study found that *Alteromonas* significantly reduced the expression of genes involved in nitrogen metabolic pathways under carbon and iron colimitation (78).

The stimulation of inorganic nitrogen uptake in these studies is entirely consistent with our observations of *Alteromonas* and other marine heterotrophs in N-limited medium. Like E. coli, carbon-limited *Alteromonas* may be deprived of the necessary α-ketoglutarate for the assimilation of ammonia or nitrate. Alternatively, or in addition, carbon limitation may deprive the cells of the energy needed to drive the transport of these substrates. In either case, the provision of organic carbon by *Prochlorococcus* appears to satisfy the requirements for enhanced inorganic nitrogen uptake and assimilation by these heterotrophs, outcompeting *Synechococcus* in the process.

Previous studies have highlighted the beneficial effects of heterotroph interactions with picocyanobacteria (40–42, 59, 79–82). Previously, we described how heterotrophic bacteria protect *Prochlorococcus* from oxidative stress (12, 38). Coe et al. (83) and Roth-Rosenberg et al. (84) have shown that heterotrophs promote the survival of *Prochlorococcus* during long-term light and nutrient (N or P) deprivation, respectively. Christie-Oleza et al. (59) found a similar relationship between *Synechococcus* and a marine roseobacter. In that study, long-term coexistence under nutrient limitation was facilitated by an exchange of resources between the phototroph and heterotroph.

Interactions between picocyanobacteria have been less well characterized, but a recent study by Knight and Morris (85) showed that *Synechococcus* could aid the growth of *Prochlorococcus* under conditions simulating ocean acidification. The mechanism of this help was not identified, but because these cocultures were grown in the presence of *Alteromonas* sp. EZ55, the authors speculated that *Synechococcus* could help *Prochlorococcus* indirectly by stimulating EZ55. The potential for allelopathic interactions between picocyanobacteria has also been noted (86–88).

Our study provides a new dimension to picocyanobacterium-heterotroph and picocyanobacterium-picocyanobacterium interactions: the ability of one phototroph (*Prochlorococcus*) to drive a shift from coexistence to competition between a second phototroph (*Synechococcus*) and a heterotroph. Christie-Oleza et al. (59) found that *Synechococcus* and heterotroph strains coexist during prolonged coculture in unamended seawater and that upon N addition, cross-feeding could occur by the conversion of N substrates unusable by the other microbe: the heterotroph strain could convert organic nitrogen (peptone) to ammonia, while WH7803 could convert nitrate to dissolved organic nitrogen. In our study, both the heterotroph and phototroph could utilize nitrate and nitrite, and unless the former was mutated in its ability to utilize these resources, the heterotroph could apparently outcompete the *Synechococcus* strain for this resource when fed organic carbon by *Prochlorococcus*.

While usually found at abundances of 10^4^ cells mL^−1^ or lower in the open ocean (89–91), *Alteromonas* was chosen as a proxy for the heterotrophic community because of previously described interactions with *Prochlorococcus*. The tripartite interaction that influenced the success of *Prochlorococcus* over *Synechococcus* is likely due to the nutrient utilization capabilities of the heterotrophic bacteria rather than an adaptation to nutrient-limited growth. However, to explore this interaction further, a future direction of this work will be to observe tripartite outcomes upon the inclusion of dominant oligotrophic heterotrophs, such as SAR11 *Pelagibacter*, to determine if these metabolic interactions occur between numerically dominant members of each trophic level (92, 93).

### Conclusion.

This study demonstrates that metabolic interactions between trophic groups can influence relative abundances within trophic groups. The prediction that *Prochlorococcus* outcompetes rival phytoplankton, including *Synechococcus*, under nutrient limitation is largely confirmed, but this outcome may require the ability of *Prochlorococcus* to energize heterotrophic bacteria to outcompete their photosynthetic rivals for resources that they themselves do not use. If our results can be extrapolated to the natural environment, they highlight an important connection between carbon and nitrogen availability and suggest that complex microbial interactions can benefit streamlined, efficient genera such as *Prochlorococcus* to the detriment of their competition.

## MATERIALS AND METHODS

### Strains and culturing.

Axenic cultures of *Prochlorococcus* strains MIT9215, MIT9312, and MED4 and *Synechococcus* strains WH7803, CC9605, and WH8102 were used in this study. Stock cultures of cyanobacteria were initially maintained in an artificial seawater medium, AMP-A (12, 94, 95), and were inoculated and serially maintained (for up to 2 years) in AMP-MN (this study) (described below) to prevent the introduction of excess nitrogen (N). The axenicity of cyanobacterial stocks and experimental cultures was tested routinely by diluting a small volume of the culture into 1/10× *Prochlorococcus* AC (ProAC; Difco) and yeast tryptone sea salts (YTSS) media and incubating these cultures in the dark at room temperature for up to 6 weeks to monitor any increase in turbidity indicating the presence of heterotrophic bacteria (35). All experiments were carried out at 24°C in I36VLX incubators (Percival, Boone, IA) with modified controllers that allowed gradual increases and decreases of cool white light to simulate sunrise and sunset, with a peak midday light intensity of 150 μmol quanta m^−2^ s^−1^ on a 14-h/10-h light/dark cycle (96). Ammonium (NH_4_^+^) was the N amendment in all experiments, unless otherwise stated, as it can be used by all strains in this study. Experiments that included different NH_4_^+^ concentrations were performed with NH_4_^+^ amendments to the AMP-A derivative AMP-MN (minus nitrogen), which is identical to AMP-A except that no N source is included. Stepwise amendments of NH_4_^+^ to AMP-MN and subsequent regression analysis of maximal *Prochlorococcus* abundances indicated that the residual N bioavailable to *Prochlorococcus* and *Synechococcus* was approximately 0.164 μM (see Fig. S1 in the supplemental material) (*R*^2^ = 0.9729).

Axenic heterotrophic bacteria utilized were Alteromonas macleodii strain EZ55 (35), Vibrio fischeri strain ES114 (97), and *Phaeobacter* sp. strain Y3F (98). Cultures of heterotrophs grown overnight were inoculated from cryopreserved stocks prior to each experiment (−80°C in YTSS plus 10% glycerol) into 5-mL volumes of YTSS (99) and incubated with shaking at 140 rpm at 24°C. Before inoculation into cyanobacterial cultures, heterotrophs were washed three times in 1.5-mL microcentrifuge tubes by centrifugation at 8,000 rpm for 2 min in a tabletop microcentrifuge and resuspension in 1 mL AMP-MN.

While all culture media were sterilized by autoclaving, sterilized spent or *Prochlorococcus*-conditioned medium was generated by culturing *Prochlorococcus* strain MIT9215 in large volumes of AMP-MN (∼300 mL). At stationary phase (25 to 30 days), these cells were removed by gentle filtration (−7 inHg) in a 1-L filter tower (Nalgene) using 0.2-μm-pore-size GTTP isopore membrane filters (MilliporeSigma, Burlington, MA). Previous studies indicated that low-pressure filtration does not cause detectable rupture of *Prochlorococcus* cells during filtration (12). The sterility of this conditioned medium was determined by flow cytometry alongside the experiments in which it was utilized, in addition to the purity assay detailed above.

### Quantification of cyanobacterium and heterotroph abundances.

The abundances of cyanobacteria were quantified by flow cytometry using a Guava EasyCyte 8HT flow cytometer (Millipore, Burlington, MA) with populations of *Prochlorococcus* and *Synechococcus* differentiated in cocultures by their red and red/yellow fluorescence, respectively (35, 100). Heterotrophs in mono- and coculture experiments were quantified by viable counting with serial dilutions on YTSS–1.5% agar plates incubated at 24°C.

### Transposon mutagenesis.

Mutants of Alteromonas macleodii strain EZ55 incapable of growing on nitrate (NO_3_^−^) as a sole N source were generated by transposon mutagenesis using a mini-Himar1 *Mariner* transposon carrying a kanamycin resistance-selectable marker (101). The RB1 plasmid vector containing the transposon was propagated in Escherichia coli strain WM3064, a *pir*^+^ and 2,6-diaminopimelic acid (DAP) auxotroph donor strain (102). Cultures of the donor strain grown overnight were inoculated from cryopreserved stocks (−80°C in LB plus 10% glycerol) into 5 mL of LB amended with 10 μg/mL of kanamycin and 150 μL of 100 mM DAP (Alfa Aesar, Haverhill, MA) and incubated with shaking at 37°C. Conjugations with EZ55 were performed by plating both the donor and recipient onto YTSS agar plates for 8 h. Exconjugants were selected on plates containing YTSS plus 10 μg/mL kanamycin. Selected colonies were screened for NO_3_^−^ utilization by replica plating (103) on AMP-A agar with 1.5% Noble agar (Difco) amended with 500 μM sodium pyruvate (Sigma-Aldrich) and either 400 μM NH_4_^+^ or 882 μM NO_3_^−^ as the nitrogen source. Replica-plated colonies growing solely on plates containing NH_4_^+^ were transferred again into tubes of AMP-A with excess carbon and different nitrogen sources to confirm that the mutants were unable to grow on nitrate or nitrite. The insertion location of the *Mariner* transposon within the *nirB* gene was verified by arbitrary PCR (104), Sanger sequencing, and BLAST comparisons with the EZ55 genome (IMG accession number 2785510739).
