# Supplementary figures and images for: Prochlorococcus Exudate Stimulates Heterotrophic Bacterial Competition with Rival Phytoplankton for Available Nitrogen
Source: mBio. 2022 Jan 11;13(1):e02571-21. doi: 10.1128/mbio.02571-21 (PMC8749424; doi:10.1128/mbio.02571-21)

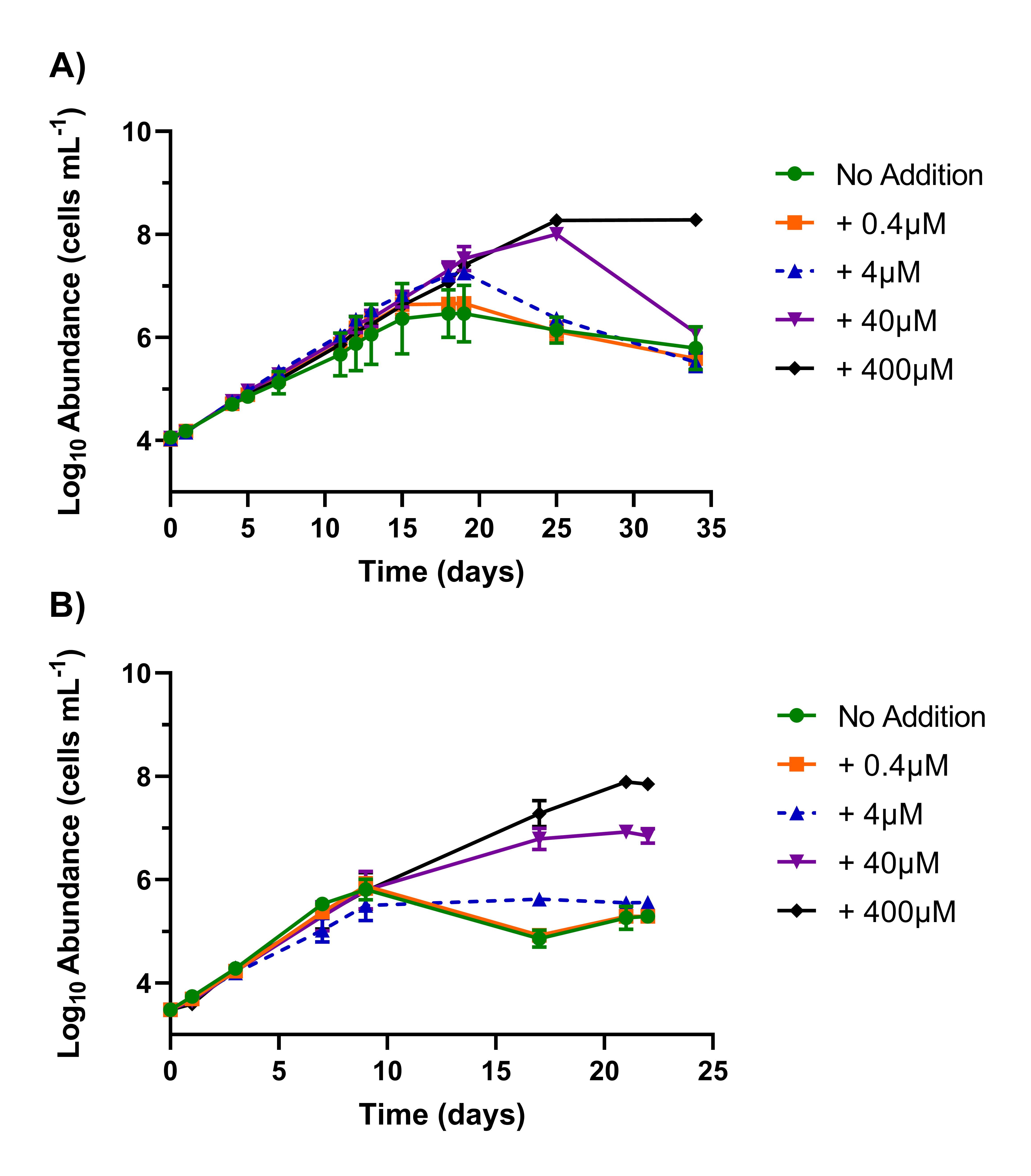

Supplement: FIG S1 [file mbio.02571-21-sf001.tif]

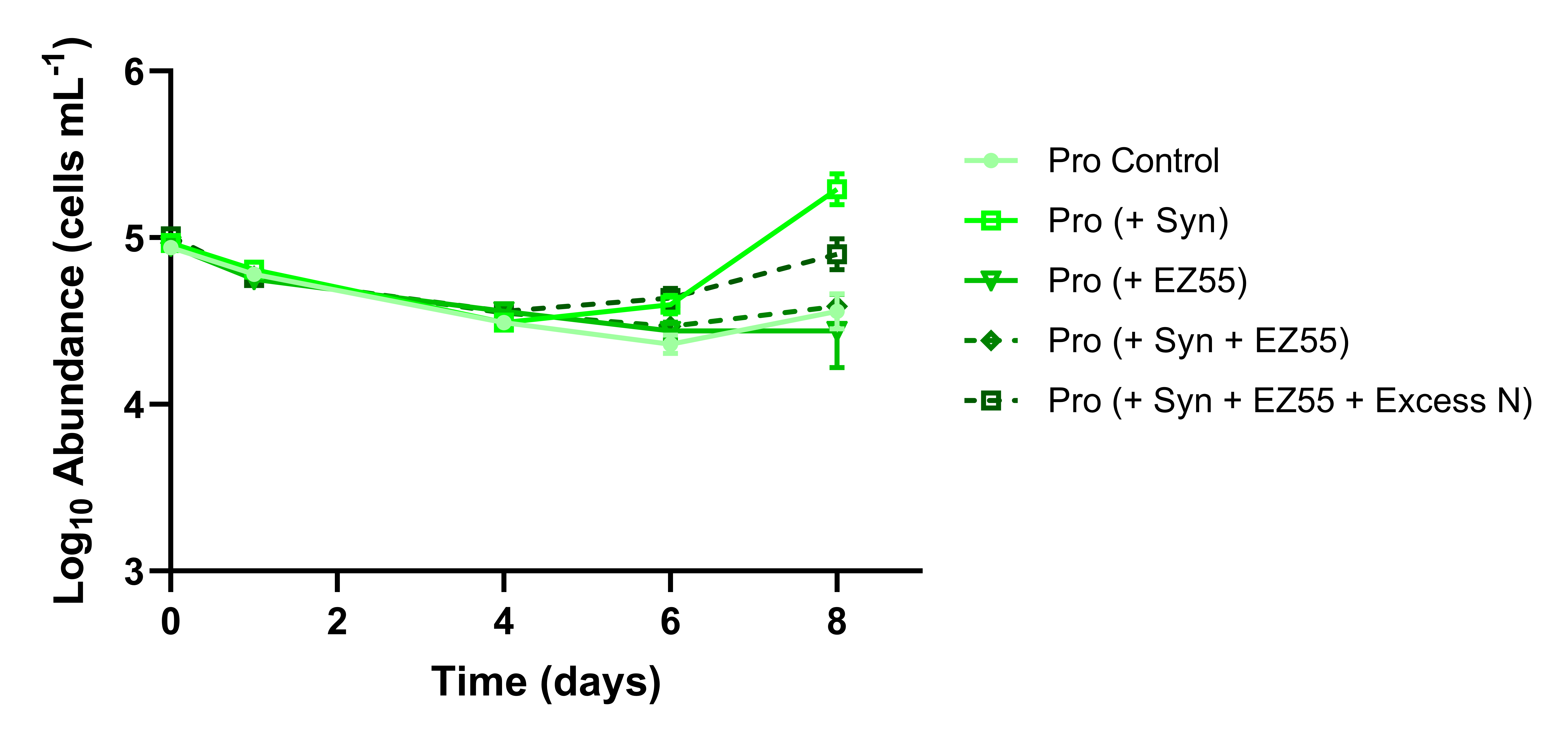

Supplement: FIG S2 [file mbio.02571-21-sf002.tif]

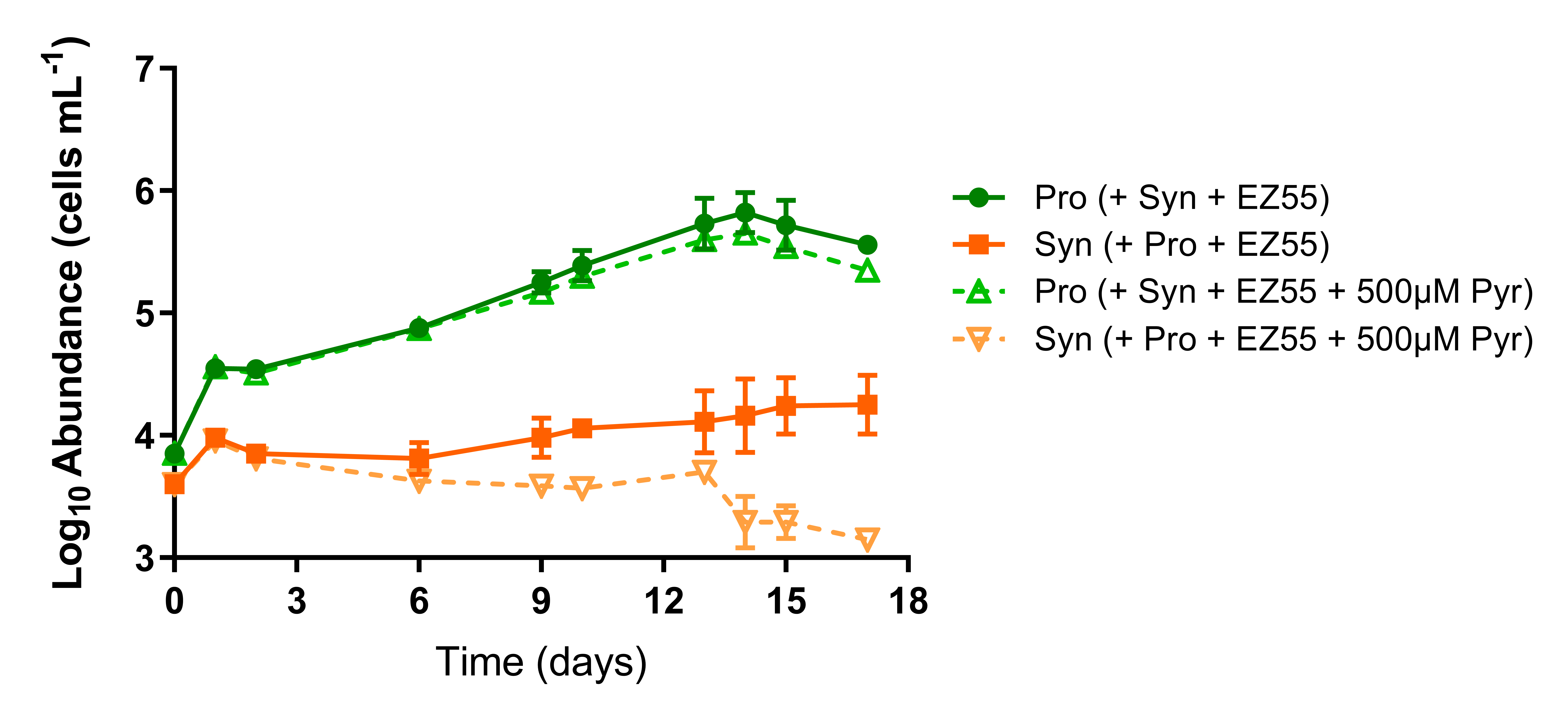

Supplement: FIG S3 [file mbio.02571-21-sf003.tif]

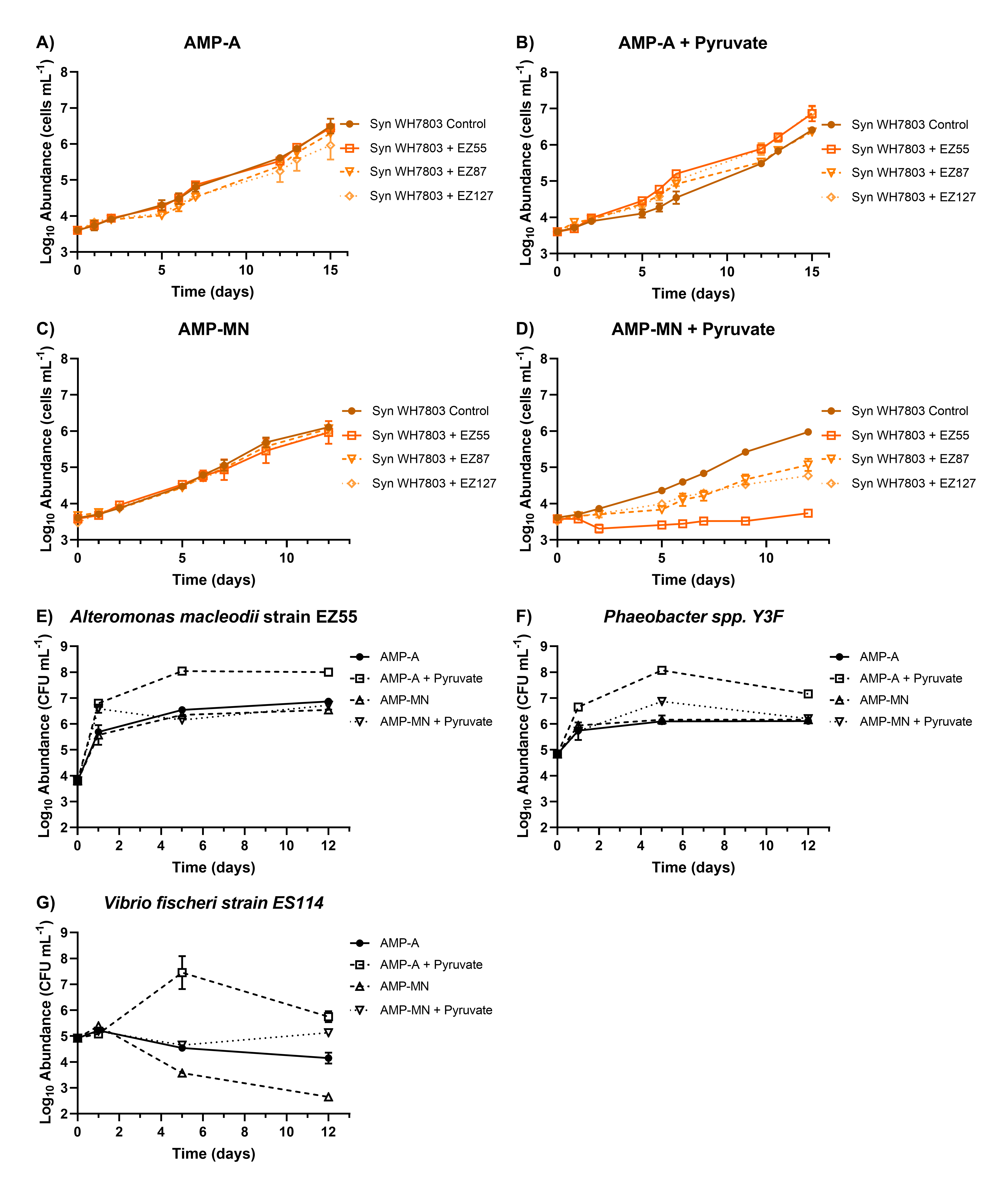

Supplement: FIG S4 [file mbio.02571-21-sf004.tif]
